# Supplementary material for: Coronarin D Induces Apoptotic Cell Death and Cell Cycle Arrest in Human Glioblastoma Cell Line
Source: Molecules. 2019 Dec 9;24(24):4498. doi: 10.3390/molecules24244498 (PMC6943529; doi:10.3390/molecules24244498)
Supplement: Supplementary file 1 [file molecules-24-04498-s001.pdf]

Coronarin D Induces Apoptotic Cell Death and Cell Cycle Arrest in Human Glioblastoma Cell Line

Yollanda E. M. Franco [1,2,*,†], Marcia Y. Okubo [3,4,†], Adriana Della Torre [3], Paula P. Paiva [3], Marcela N. Rosa [5], Viviane A. O. Silva [5], Rui M. Reis [5,6,7], Ana Lucia T. G. Ruiz [4,8], Paulo M. Imamura [9], João Ernesto de Carvalho [4,8] and Giovanna B. Longato [1,2]

1 Research Laboratory in Molecular Pharmacology of Bioactive Compounds. São Francisco University - 12916-900 Bragança Paulista, SP, Brazil; carvalho@fcf.unicamp.br (G.B.L.)
2 Posgraduate program in Health Science, São Francisco University - 12916-900 Bragança Paulista, SP, Brazil
3 Chemical, Biological and Agricultural Pluridisciplinary Research Center (CPQBA), University of Campinas – UNICAMP, 13148-218 Paulínia, SP, Brazil; yumiokuboh@gmail.com (M.Y.O.); adriana_biotec@yahoo.com.br (A.D.T.); paula.p21@gmail.com (P.P.P.)
4 Posgraduate program in dentistry, Piracicaba Dental School, University of Campinas -13 414-903, Piracicaba, SP, Brazil; ana.ruiz@fcf.unicamp.br (A.L.T.G.T.); carvalho@fcf.unicamp.br (G.E.d.C.)
5 Molecular Oncology Research Center, Barretos Cancer Hospital, Barretos, Brazil; nr.marcela2@gmail.com (M.N.R.); vivianeaos@gmail.com (V.A.O.S.); ruireis.hcb@gmail.com (R.M.R.)
6 Life and Health Sciences Research Institute (ICVS), School of Medicine, University of Minho, Braga, Portugal
7 ICVS/3B's – PT Government Associate Laboratory, Braga/Guimarães, Portugal
8 Faculty of Pharmaceutical Sciences, University of Campinas, UNICAMP, 13081-970 Campinas, SP, Brazil
9 Institute of Chemistry, University of Campinas-UNICAMP, P.O. Box 6154, 13083-970, Campinas, SP, Brazil; imam@iqm.unicamp.br (P.M.I.)
* Correspondence: yollanda.moreira@hotmail.com; Tel.: +55- 11- 996672895

† These authors contributed equally to this work.

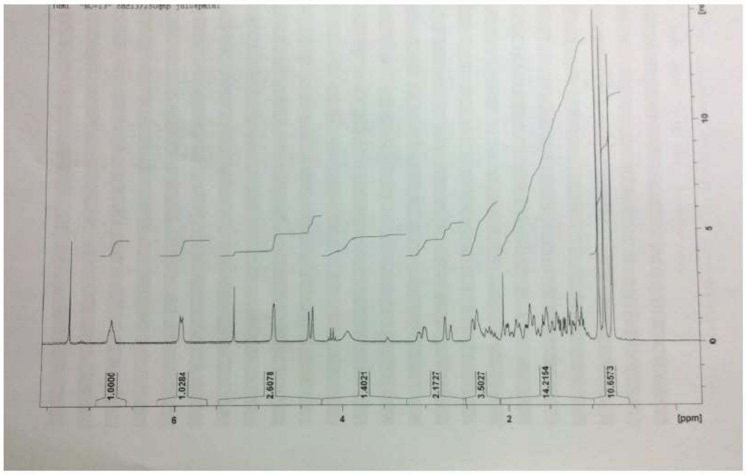

Figure 1. Coronarin D ^{1}H-NMR spectra.

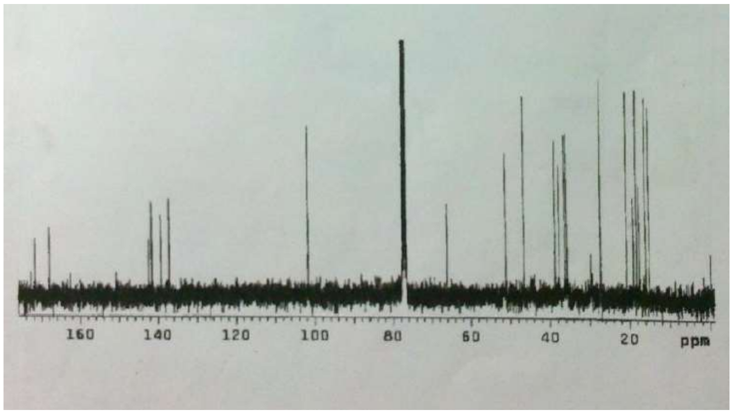

Figure 2. Coronarin D ^{13}C-NMR spectra.

Table S1. Coronarin ^{13}C-NMR spectral data.

Carbon	Coronarin D
1	392
2	193
3	42,0
4	335
5	553
6	241
7	378
8	1481
9	561
10	394
11	255
12	1436
13	1245
14	535
15	965
16	1707
17	1076
18	335
19	217
20	143
